# Myctobase, a circumpolar database of mesopelagic fishes for new insights into deep pelagic prey fields

**DOI:** 10.1038/s41597-022-01496-y

**Published:** 2022-07-13

**Authors:** Briannyn Woods, Rowan Trebilco, Andrea Walters, Mark Hindell, Guy Duhamel, Hauke Flores, Masato Moteki, Patrice Pruvost, Christian Reiss, Ryan A. Saunders, Caroline Sutton, Yi-Ming Gan, Anton Van de Putte

**Affiliations:** 1 Institute for Marine and Antarctic Studies, University of Tasmania, Hobart, 7004 Australia; 2 CSIRO, Oceans and Atmosphere, Hobart, 7004 Australia; 3 Laboratoire BORA (UMR 8067), Muséum National d’Histoire Naturelle, Paris, 75005 France; 4 Alfred-Wegener-Institut Helmholtz-Zentrum für Polar und Meeresforschung (AWI), Bremerhaven, 27570 Germany; 5 Tokyo University of Marine Science and Technology, Tokyo, 108-8477 Japan; 6 National Institute of Polar Science, Tokyo, 190-8518 Japan; 7 Antarctic Ecosystem Research Division, Southwest Fisheries Science Center, La Jolla, California 92037 USA; 8 British Antarctic Survey, Cambridge, CB3 0ET UK; 9 Royal Belgian Institute for Natural Sciences, Brussel, B-1000 Belgium; 10 Université Libre de Bruxelles, Brussel, B-1000 Belgium

**Keywords:** Ecosystem ecology, Biodiversity, Biogeography, Ecological modelling

## Abstract

The global importance of mesopelagic fish is increasingly recognised, but they remain poorly studied. This is particularly true in the Southern Ocean, where mesopelagic fishes are both key predators and prey, but where the remote environment makes sampling challenging. Despite this, multiple national Antarctic research programs have undertaken regional sampling of mesopelagic fish over several decades. However, data are dispersed, and sampling methodologies often differ precluding comparisons and limiting synthetic analyses. We identified potential data holders by compiling a metadata catalogue of existing survey data for Southern Ocean mesopelagic fishes. Data holders contributed 17,491 occurrence and 11,190 abundance records from 4780 net hauls from 72 different research cruises. Data span across 37 years from 1991 to 2019 and include trait-based information (length, weight, maturity). The final dataset underwent quality control processes and detailed metadata was provided for each sampling event. This dataset can be accessed through Zenodo. *Myctobase* will enhance research capacity by providing the broadscale baseline data necessary for observing and modelling mesopelagic fishes.

## Abstract (summary)

**Table:** 

Measurement(s)	Abundance • Standard length • Wet weight
Technology Type(s)	Trawl net • ruler-based body length measuring method • Motion compensated balance
Sample Characteristic - Organism	Actinopteri • Cephalopoda
Sample Characteristic - Environment	oceanic pelagic zone biome
Sample Characteristic - Location	Southern Ocean

## Background & Summary

Open-ocean pelagic ecosystems are under-represented in databases of marine biodiversity, despite holding the largest biomass of organisms on Earth^1^. The open-ocean pelagic community predominantly consists of fish, crustaceans and cephalopods that inhabit mesopelagic (200–1000 m) and bathypelagic (1000 m to >4000 m) depths^2^. Many pelagic species undertake diel vertical migration (DVM), moving from the depths to shallower waters at dusk and in the reverse direction at dawn, possibly constituting the largest animal migration on the planet^3^. This has implications for carbon sequestration and climate regulation, as organisms actively transport organic carbon from the surface to the deep ocean^3–6^.

Mesopelagic fishes are a central component of open-ocean pelagic communities dominating global vertebrate biomass with estimates of up to 10 billion tons^4,7,8^. They are thought to represent a key link to coupling physical-biogeochemical models to the population dynamics of top-predators^6,9^. Thus, the open-ocean pelagic environment and its inhabitants are critical components for the provision of globally important ecosystem services^1,2^.

In the Southern Ocean, mesopelagic fishes are key prey for sentinel species such as seals and seabirds^10–12^. As major consumers of secondary productivity (zooplankton) they also exert control on lower trophic levels of oceanic food webs^13,14^. Despite their ecologically important role, there are gaps in our knowledge of their biodiversity, abundance, biomass, and the processes that shape their distribution, life cycles and behaviour^4,8,15–19^. Mesopelagic fishes are difficult to sample due to their patchy distribution and ability to avoid and escape pelagic nets^8,20,21^. This is confounded by the differences in catch efficiency between gear types, leading to biased estimates of abundance and biomass^4,8^.

Mesopelagic fishes are likely to be impacted by ocean-warming with evidence indicating future range shifts of temperate species, range reductions of Antarctic species^16,22^ and possible biogeographic shifts in body size patterns^23^. This will have implications for the predators that feed upon them^14^ and potentially more broadly for global biogeochemical cycles^24^. Tracking the future distribution and abundance of species requires the development of reliable population baseline estimates and an understanding of the biases associated with different sampling strategies^25–28^.

The advent of biodiversity informatics has seen the development of open-access biodiversity data repositories, such as the Global Biodiversity Information Facility (GBIF) and the Ocean Biogeographic Information System (OBIS), to enhance research output in the context of a global biological monitoring system^29^. However, much of the data for mesopelagic fishes archived in these repositories are for species occurrence only, without information on abundance or biomass. Often, the methodological metadata (such as net-type or depth of trawl) are also lacking, further limiting the scope of the analyses^1,30^.

**Fig. 1:**
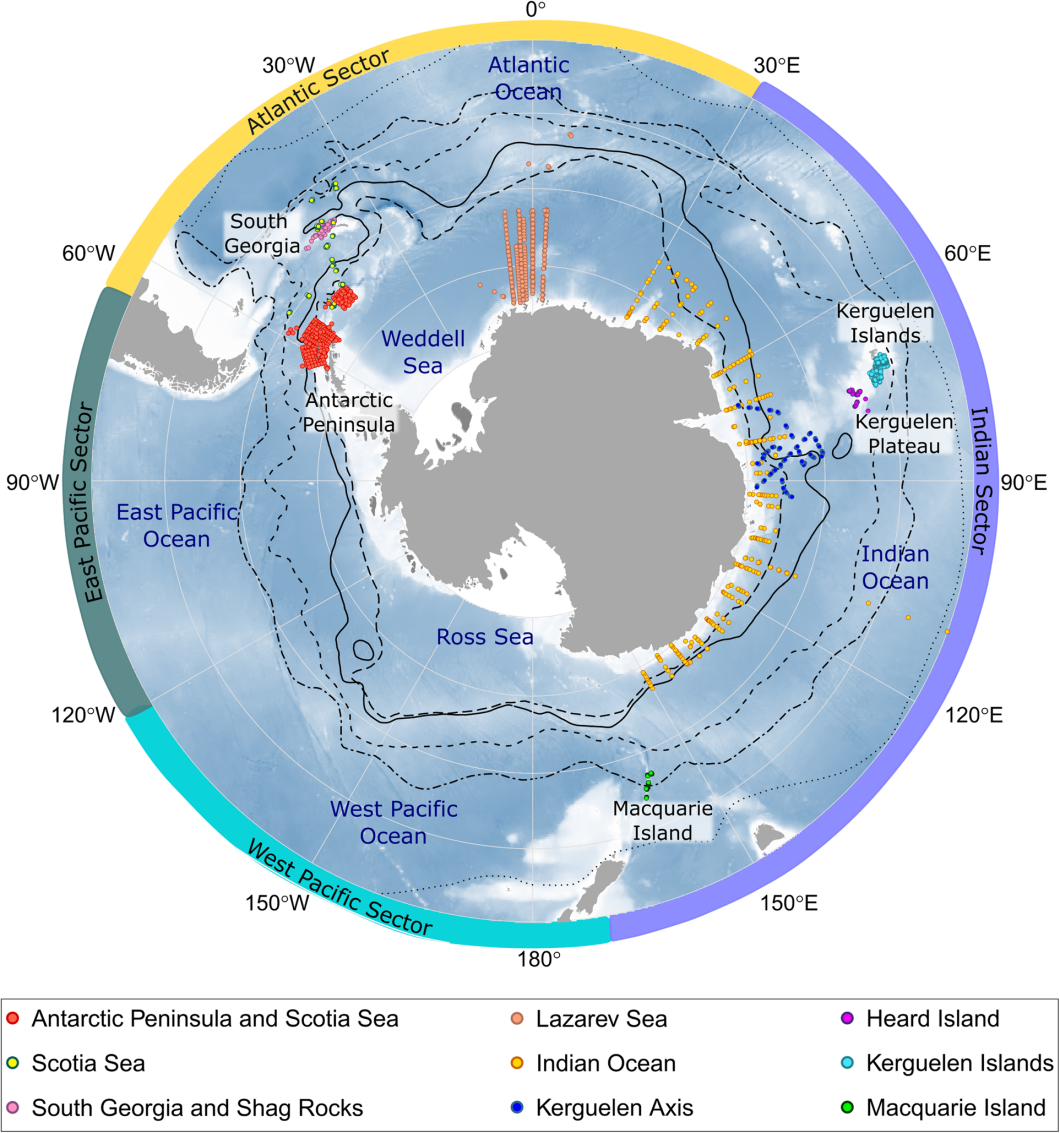
The sampling locations of mesopelagic fish trawl stations that are currently held in *Myctobase*. Trawl stations are colour coded to correspond to the location listed in Online-only Table 1 (colour key is indicated within the black box). Black lines indicate mean frontal positions^52^. Starting from the outer line, frontal features are as follows: Subtropical Front (dotted line); Subantarctic Front (dash-dot line); Polar Front (dashed line); southern Antarctic Circumpolar Current Front (solid line); southern boundary of the ACC (long dashed line). Numbered labels around the outside of the map indicate the longitude. Each latitude line represents 10° of latitude where 75° S is at the Antarctic continent and 40° S is at the external line.

Decades of data from net sampling of the pelagic environment exist for localised regions of the Southern Ocean as part of multiple national Antarctic research programs^31^. Here, we take the important step of integrating and standardising the format of published and unpublished survey data on the abundance, biomass, biodiversity, and methodological metadata for mesopelagic fishes of the Southern Ocean. We collected and synthesised data from the Scotia Sea^14,32–34^, the Antarctic Peninsula^35,36^, the Lazarev Sea^37,38^, the Kerguelen Plateau^39–43^, East Antarctica^44^, Macquarie Island^45,46^ and unpublished data from the Indian Ocean from Japan’s national Antarctic program into a single dataset called *Myctobase* (Fig. 1).

The long-term aims of *Myctobase* are (1) to provide a single findable, standardised, and citable resource for multiple combined datasets; (2) to provide the baseline data upon which to measure the status and future trends of the mesopelagic fish assemblage; (3) to support further research such as investigating the patterns and biophysical drivers of diversity; and (4) to provide the broadscale spatiotemporal perspective necessary for the holistic management and conservation of the open-ocean pelagic ecosystem in the Southern Ocean. The dataset is available on GBIF, OBIS and Zenodo to support the management of the open-ocean pelagic ecosystem. Researchers wishing to contribute to this project should contact the corresponding authors. We hope that this database will continue to grow, providing a resource for ongoing monitoring of the Southern Ocean pelagic ecosystem.

## Methods

### Sampling design

Samples were collected onboard multiple national Antarctic research cruises between the years 1991–2016 and for the year 2019 and jointly covered all calendar months (Online-only Table 1). Net sampling occurred predominantly in the Indian and Atlantic Sectors of the Southern Ocean (Fig. 1).

Mesopelagic fishes were sampled using a range of gear types as a “standard” sampling gear does not currently exist^21^. Samples were collected from pelagic trawls using opening-closing net systems with a range of Rectangular Midwater Trawl (RMT) and International Young Gadoid Pelagic Trawl (IYGPT) nets, an Isaacs-Kidd Midwater Trawl (IKMT) net and a Matsuda-Oozeki-Hu Trawl (MOHT) net (Table 1, Online-only Table 2). Opening-closing net systems allowed for the sampling of discrete depth intervals and included stratified oblique and horizontal trawls. Survey designs (for trawl locations) included randomised design, designated stations, and target trawls on acoustically detected aggregations of fish (see Online-only Table 1 for more information on sampling methodology).

**Table 1:** Data in *Myctobase* were collected with the following net types which are commonly referred to by their associated acronyms.

Net Type	Acronym
Rectangular Midwater Trawl net	RMT
International Young Gadoid Pelagic Trawl net	IYGPT
International Young Gadoid Pelagic Trawl with Mid-water Open Close net	IYGPT with MIDOC net
Matsuda-Oozeki-Hu Trawl net	MOHT
Isaacs-Kid Midwater Trawl net	IKMT

Once onboard, samples were sorted to the lowest taxonomic level possible using published guides^41,47^ and personal/institutional reference collections. Taxonomic identities were verified in home laboratories. Standard length (mm) and wet weight (g) measurements were taken onboard with a motion compensated balance or in home laboratories. Samples were preserved in either ethanol, formalin or frozen for further analyses.

Detailed information on methodologies utilised for each research cruise can be found in the relevant citations listed in Online-only Table 1.

### Data collection and processing

Potential data holders were identified by compiling a metadata catalogue of scientific publications and existing survey data for Southern Ocean mesopelagic fishes. Data holders were invited to contribute published and unpublished data to the Myctobase project. A standardised template for the collation of multiple datasets was created using Darwin Core terms^48^ where possible. The terms used in the template alongside definitions for each term can be found in Table 1. Data were collated with R statistical software, version 4. 0. 4^49^.

**Fig. 2:**
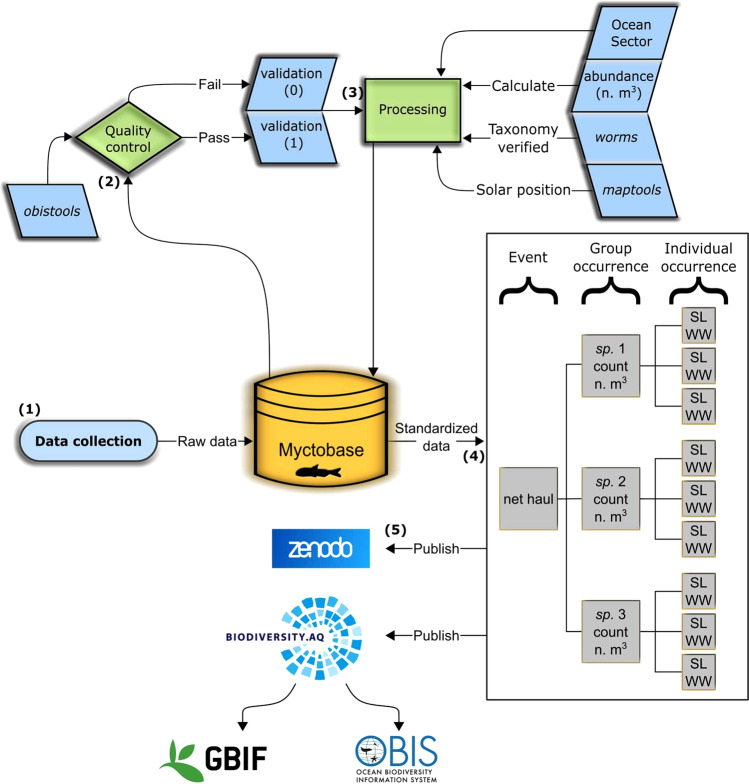
Schematic illustrating the quality control and processing steps leading to the standardised data output of *Myctobase*. The standardised data are made available through Zenodo, the Antarctic Biodiversity Portal, the Global Biodiversity Information Facility (GBIF) and the Ocean Biogeographic Information System (OBIS). Abbreviations under ‘Individual occurrence’ are standard length (SL) and wet weight (WW).

Units of measurement were standardised across datasets. Length and weight measurements were converted into millimetres and grams, respectively. The taxonomy of each observation was verified and associated aphia IDs (globally unique and stable identifiers for each taxonomic name) were retrieved from the World Register of Marine Species using the R package, WoRMS^50^ (Fig. 2).

Abundance was calculated for each net haul by species per filtered volume of water. This was performed by dividing the species count (n) by the volume filtered (m^3^). This was not possible for all net hauls and species as count and volume filtered information were not always recorded or technical difficulties were encountered during the research cruise. Methodology for calculating volume filtered varied between datasets. Several cruises used mechanical flow meters while others used a calculation where the nets filtering area (m^2^) was multiplied by the distance it was towed (m). The filtering area of the MOHT net was calculated by multiplying the net mouth area by a coefficient estimated from calibration tows in calm seas. The coefficient was estimated under a calm sea condition using a net frame with a flowmeter which was vertically deployed up to 100 m wire out. The net frame was retrieved slowly to the sea surface and a count from the flowmeter was recorded. This procedure was repeated five times and an average value was calculated to obtain the coefficient. For some datasets, volume filtered was not previously calculated, but the information needed to calculate the volume filtered had been recorded. In these instances, we retrospectively calculated the volume filtered using this information. This was shown in a separate column to the values that were obtained at the time of a cruise, this is labelled *volumeFiltered2* in the database. Detailed information on the methodologies utilised to obtain volume filtered, including our retrospective calculations, for each cruise can be found in Online-only Table 2.

The solar position and day/night information was added for each observation based on the date, time and the start latitude and longitude of each net haul using the R package, *maptools*^51^ (Fig. 2). Solar position is the angle of the sun in relation to the horizon (0°), thus dawn was defined as a solar position of −12° to 12° before midday, and dusk was defined as a solar position of 12° to −12° after midday. Day was defined as the period after dawn and before dusk and night was defined as the period after dusk and before dawn. We added the zone (eg. Northern, Subantarctic and Antarctic) in which net hauls were undertaken using the definitions in^52^. The sector in which the net haul was undertaken was additionally added following definitions in^53^, which define four major sectors: Atlantic, Indian, West Pacific and East Pacific (Fig. 2).

## Data Records

The dataset is comprised of three comma-separated files which are freely available at Zenodo^54^. Filenames adhere as closely as possible to the naming convention set out by the Darwin Core Standard^48^. The first file (event.csv) describes the survey methodology. Each row has its own unique event ID, which consists of the institute, cruise, event number (as recorded in the voyage logbook) and the net number (institute_cruise_event_net). An event ID represents the sampling event or net haul and contains the details of the event including date, time, position (latitude, longitude, and depth), sampling protocol, net type, net mesh size, tow speed, volume filtered and haul type (station, routine, target, test, surface or failed). The second file (groupOccurrence.csv) contains the catch data linked to the survey methodology by an event ID. Each row has its own unique occurrence ID, which is the event ID and aphia ID (retrieved from WoRMS) combined (eventID_aphiaID). An occurrence ID contains taxonomic information (eg. phylum, class, order, and family), the number of individuals (n) and estimated abundance (n_m^3^) for the associated sampling event. The final file (individualOccurrence.csv) contains measurements of individuals. Each row contains the event and occurrence ID, which links each measurement to the first and second file, and a ‘catalogNumber’, linking the data to the original dataset for traceability. Rows also contain taxonomic information, standard length (mm), weight (g), life stage and reproductive maturity of the individual (following definitions in^55^) and sex, where available. Additional notes on the preservation method and whether the specimen was measured before or after preservation are included. The presence of NA values in *Myctobase* are indicative of missing data. See associated metadata record for definitions and units for each variable (Supplementary Table 1). Citations for all datasets held in *Myctobase* can be found in Online-only Table 1.

### Spatial and temporal coverage

*Myctobase* currently holds 4780 net hauls from 3775 sampling stations and 72 different research cruises from across the Southern Ocean. There are currently 17,491 occurrence records in *Myctobase*.

The highest concentration of data points is within the Atlantic Sector (45% of net hauls) spanning from the Antarctic continent to the Polar Front at 50° S (Fig. 1). The East and West Pacific Sector stands as a major gap in the spatial coverage of *Myctobase* (21% and 1% of net hauls in the East and West, respectively).

Vertically, data extend from the surface down to a maximum depth of 2000 m. More than half of the trawls took place in the epipelagic layer at 0–200 m (n = 3713). Data from the mesopelagic (200–1000 m) and bathypelagic (>1000 m) layers were also recorded from stratified oblique trawls.

Data span from 1991–2016 and 2019, with no data for 2017 and 2018 (Online-only Table 1). The year 2006 contains the highest number of net hauls (11%). Cumulatively, the datasets within *Myctobase* cover data for every month (NB. not every month of every year), with 76% of net hauls occurring in the summer months (November to March).

### Records of occurrence

**Fig. 3:**
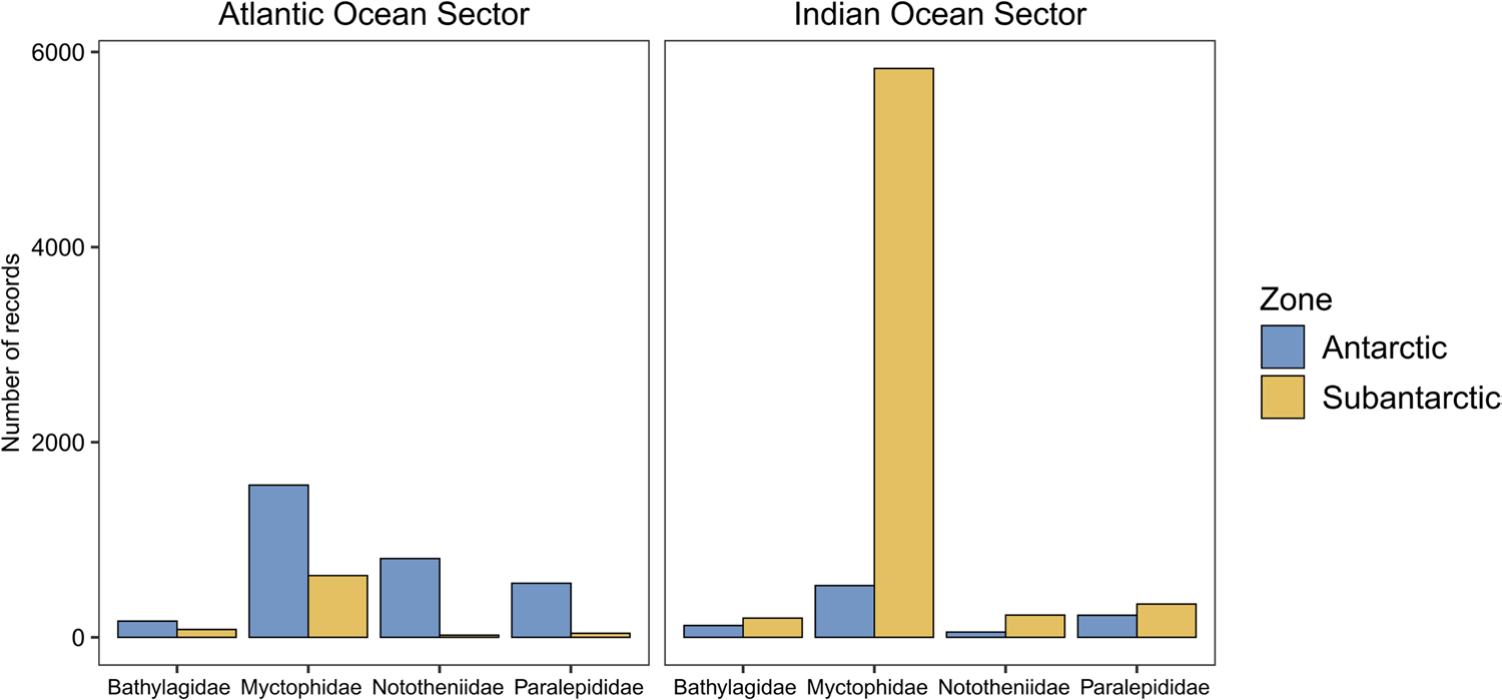
The number of records of occurrence for the four most abundant fish families in the groupOccurrence data record of *Myctobase*. Data are divided into Ocean Sectors (Atlantic and Indian) and zones (Antarctic and Subantarctic) of the Southern Ocean. Zones of the Southern Ocean are defined by^52^.

The four most abundant fish families in *Myctobase* are Paralepididae, Nototheniidae, Myctophidae and Bathylagidae. Myctophidae make up the highest number of records in *Myctobase* for both the Atlantic and Indian sectors (Fig. 3). In the Indian Ocean sector, the highest number of records for Myctophidae are in the Subantarctic zone. Conversely, in the Atlantic Ocean sector the highest number of records for Myctophidae are in the Antarctic zone. There are a similar number of records for Bathylagidae, Nototheniidae and Paralepididae in the Indian sector for both the Antarctic and Subantarctic zones. In the Atlantic Ocean sector, the highest number of records for Bathylagidae, Nototheniidae and Paralepididae are in the Antarctic zone.

## Technical Validation

The final dataset was subject to quality control and validation processes. Rather than removing ambiguous or incomplete records altogether, two extra columns were added to the event information (event.csv). The first column labelled *validation* is an index used to indicate whether the record passed quality control measures (0 = fail, 1 = pass). The second column labelled *validationDescription* details the reasons for a failure. Indexing the data in this way prevents the loss of potentially valuable data. For example, some events are missing latitude and longitude information, however a more general sampling location is often available and may be enough for regional analyses (Fig. 2).

The R package *obistools*^56^ was used to validate sampling locations and dates of events. Sampling locations on land or at depth values higher than the bathymetry raster used in *obistools* did not pass the quality control step and thus were given a ‘0’ in the *validation* column. Further, records missing latitude, longitude and date/time data were similarly identified. Records where technical difficulties were recorded, such as net failing to open or close were also allocated a ‘0’ in the *validation* column (Fig. 2).

Abundance was standardised to number of individuals per m^3^ (n_m^3^) for all datasets. In some instances, abundance could not be calculated due to missing count or volume filtered data. Additionally, abundances were not calculated for *US AMLR Program* as this data were considered bycatch and not the sole focus of the sampling program. Subsequently, this data is important for documenting the species observed and overall distribution in the region of the Antarctic Peninsula. Important information regarding the use of abundance values is available in ‘Usage Notes’.

Although the purpose of the dataset is to document the occurrences and relevant metadata of mesopelagic fish species, we also retained records of cephalopods. Occurrence records of cephalopods alongside those of fish were included in some of the datasets contributed to *Myctobase*. Cephalopods are similarly important species of the open-ocean pelagic community and are a key group supporting the flow of energy from primary producers to higher order predators in Southern Ocean food webs^57–59^. Data on squid are limited due to their low catchability with scientific nets^59^. As such, we retained records to maximise availability of valuable data. The data are structured to enable users to easily filter out cephalopod occurrences if they are not of interest.

Taxonomic fields within the final dataset were checked for spelling errors and to verify the usage of valid/accepted names according to WoRMS. Where appropriate, records were corrected. All original data records were archived for future data checking and validation. Users can provide feedback for any data record to the corresponding authors.

## Usage Notes

*Myctobase* will enhance research capacity by facilitating international effort toward observing and modelling mesopelagic fish taxa. The data held in *Myctobase* are suitable for a number of applications, for example, investigating the biophysical determinants shaping patterns of occurrence and biodiversity. Further examples of data use can be found in the citations listed in Online-only Table 1. Data can be analysed using a variety of statistical software such as R^49^ or Matlab^60^.

*Myctobase* contains data from 72 different research cruises across the Southern Ocean. Each cruise employed a specific sampling strategy and provided varying levels of detail on methodology and samples collected. The different sampling methodologies used between datasets necessitates caution when comparing abundance estimates, as well as length and weight measurements across research cruises. The biases associated with net type and mesh size of the net have previously been described^8,21^, and are likely to influence the final output of analyses that use data from different projects. For example, the IYGPT has a larger mesh size at the front of the net limiting the size of fish that may be caught due to smaller fish escaping through the mesh. The minimum size limit has been suggested to be approximately 25–35 mm^61^. This suggests that nets with larger mesh sizes may not be appropriate for calculating densities of species that can escape the net. However this is a complex issue which requires further study as the net will also exert a herding effect on fish^32^. Biases should be considered and acknowledged. *Myctobase* provides detailed information for each sampling event to ensure that researchers can account for these differences in their analyses or to enable comparison of data using similar methods.

Further, abundance values should be treated as relative rather than absolute values due to the patchy distribution of species and the limited spatial and temporal coverage of sampling creating uncertainty around estimates^4,8^. Data are best used to demonstrate the community composition, distribution, and occurrence of fish species, life history and relative abundance within the Southern Ocean. For example, there are a multitude of available modelling techniques which may be used to predict a species geographic distribution in relation to environmental variables^62,63^. Furthermore, this information provides the data necessary for ground-truthing acoustic data^64^.

We anticipate that *Myctobase* will continue to grow into a fully circumpolar database with continued collaboration from across the scientific marine community. We invite researchers to contact the corresponding author(s) to contribute data (particularly from under-represented regions, taxa, and data types such as abundance data), including data from unpublished research which can be embargoed until publication.

### Terms of use

The database is released under a CC-BY license. Users are encouraged to formally cite the data record used according to the standards and format of the journal in which they are published.

## Supplementary information

Supplementary Table 1

## Data Availability

We used freely available code from the following packages: *maptools*^51^, *obistools*^56^ and *WoRMS*^50^

## Online-only Tables

**Online-only Table 1:** Summary information including location, date, number of hauls and sampling stations, data contact and the relevant citations (ref) for detailed methodological information and individual datasets that are held in Myctobase.

Project title (cruise code)	Location	Cruise period	No net hauls	No stations	Net type	Custodian (affiliation): Contact	Method ref	Data ref	Samples available
JB11	South Georgia	1991/01/11 - 1991/02/17	28	3	RMT25	Ryan Saunders (BAS): ryaund@bas.ac.uk	Piatkowski *et al.*^32^	Collins *et al.*^65^	No
JR100	South Georgia, Shag Rocks shelves	2004/03/09 - 2004/04/05	79	71	RMT25, RMT8, IYGPT	Ryan Saunders (BAS): ryaund@bas.ac.uk	Collins *et al.*^33^	Collins *et al.*^65^	No
Discovery 2010 (JR161)	Scotia Sea	2006/10/24 - 2006/12/03	109	10	RMT25	Ryan Saunders (BAS): ryaund@bas.ac.uk	Collins *et al.*^34^	Collins *et al.*^65^	Yes
Discovery 2010 (JR177)	Scotia Sea	2007/12/30 - 2008/02/16	81	11	RMT25, RMT8	Ryan Saunders (BAS): ryaund@bas.ac.uk	Collins *et al.*^34^	Collins *et al.*^65^	Yes
Discovery 2010 (JR200)	Scotia Sea	2009/03/11 - 2009/04/18	51	10	RMT25, RMT8	Ryan Saunders (BAS): ryaund@bas.ac.uk	Collins *et al.*^34^	Collins *et al.*^65^	Yes
BROKE-EAST (brokeeast)	Indian Sector of the SO	1996/01/19 - 1996/03/31	164	130	RMT8	Australian Antarctic Data Centre	Hoddell *et al.*^66^	Hoddle *et al.* (2016)	No
SS199901	Macquarie Island	1999/01/21 - 1999/01/31	64	14	IYGPT with MIDOC trawl net	Caroline Sutton (CSIRO): Caroline.Sutton@csiro.au	Sutton *et al.*^46^; Flynn & Williams^45^	Sutton *et al.*^46^	Yes
HIPPIES	Heard Island	2003/12/08 - 2004/02/13	133	37	RMT8, IYGPT	Mark Hindell (IMAS): mark.hindell@utas.edu.au	Constable *et al.*^67^	Constable *et al.*^67^	No
BROKE-WEST (brokewest)	Indian Sector of the SO	2006/01/01 - 2006/03/01	147	125	RMT1+8	Anton Van de Putte (RBINS): avandeputte@naturalsciences.be	Van de Putte *et al.*^44,68^	Van de Putte *et al.*^44,68^	No
KAXIS	Kerguelen Axis	2016/01/11 - 2016/03/12	219	36	IYGPT with MIDOC trawl net	Rowan Trebilco (CSIRO): Rowan.Trebilco@csiro.au; Andrea Walters (IMAS): andrea.walters@utas.edu.au	Trebilco *et al.*^42^; Flynn *et al.*^69^	NA	Yes
ANT21_4_PS65 (PS65)	Lazarev Sea	2004/03/03 - 2004/05/05	206	103	RMT1+8	Hauke Flores (AWI): Hauke.Flores@awi.de	Flores *et al.*^37^; Flores *et al.*^38^	NA	No
Lakris Summer (PS69_4)	Lazarev Sea	2005/11/19 - 2006/01/12	113	113	RMT8	Hauke Flores (AWI): Hauke.Flores@awi.de	Flores *et al.*^38^	NA	No
Lakris Winter (PS69_6)	Lazarev Sea	2006/06/17 - 2006/08/21	179	84	RMT1+8	Hauke Flores (AWI): Hauke.Flores@awi.de	Flores *et al.*^38^	NA	No
KARE 22 (UM-18-08)	Indian Sector of the SO	2019/01/09 - 2019/01/15	70	10	MOHT	Masato Moteki (TUMSAT): masato@kaiyodai.ac.jp	Oozeki *et al*.^70^	NA	Yes
Ichtyoker (Ichtyo#)	Kerguelen Islands	1998 - 2000	828	828	IYGPT	Guy Duhamel (MNHN): guy.duhamel@mnhn.fr; Patrice Pruvost: patrice.pruvost@mnhn.fr	Duhamel *et al.*^41^	NA	Yes
US AMLR Program (AMLR#)	Antarctic Peninsula	1992 - 2015	2234	2116	IKMT	Christian Reiss (NOAA): christian.reiss@noaa.gov	Loeb* et al.*^35^; Reiss *et al.*^36^	NA	No

**Online-only Table 2:** Methodological information including net type and method for estimating volume filtered for each of the datasets held in Myctobase.

Project title/Cruise code	Net type	Volume filtered method	(1) Distance towed	(2) Filtering area
JB11	RMT25	Volume Filtered = (1) Distance towed (m) × (2) the net’s filtering area (m^2^)		RMT: Theoretical net area (25 m^2^)
JR100	RMT25, RMT8, IYGPT	Volume Filtered = (1) Distance towed (m) × (2) the net’s filtering area (m^2^)	Ship’s GPS position at the start and end of trawl	RMT: Theoretical net area (25 or 8 m^2^ for RMT25 and RMT8, respectively); IYGPT: theoretical height × theoretical wingspread (7 × 12 m)
Discovery 2010: JR161	RMT25	Volume Filtered = (1) Distance towed (m) × (2) the net’s filtering area (m^2^)	Ship’s GPS position at the start and end of trawl	RMT: Theoretical net area (25 or 8 m^2^ for RMT25 and RMT8, respectively)
Discovery 2010: JR177	RMT25, RMT8	Volume Filtered = (1) Distance towed (m) × (2) the net’s filtering area (m^2^)	Ship’s GPS position at the start and end of trawl	RMT: Theoretical net area (25 or 8 m^2^ for RMT25 and RMT8, respectively)
Discovery 2010: JR200	RMT25, RMT8	Volume Filtered = (1) Distance towed (m) × (2) the net’s filtering area (m^2^)	Ship’s GPS position at the start and end of trawl	RMT: Theoretical net area (25 or 8 m^2^ for RMT25 and RMT8, respectively)
BROKE-EAST	RMT8	unknown	unknown	unknown
SS199901	IYGPT with MIDOC trawl net	Volume Filtered = (1) Distance towed (m) * (2) the net’s filtering area (m^2^)	Ship’s GPS position at the start and end of trawl	theoretical height × theoretical wingspread (15 × 3.8)
HIPPIES	IYGPT	Volume Filtered = (1) Distance towed (m) * (2) the net’s filtering area (m^2^)	Ship’s GPS position at the start and end of trawl	theoretical height × theoretical wingspread
	RMT 8	Volume Filtered = (1) Distance towed (m) * (2) the net’s filtering area (m^2^)	Ship’s GPS position at the start and end of trawl	Steps:A) $\sqrt{{a}^{2}+{b}^{2}}$=oblique distance
				*a* is distance towed; *b* is vertical tow height.
				B) Oblique distance × net mouth area = filtering area
BROKE-WEST	RMT1 + 8	unknown	unknown	unknown
KAXIS	IYGPT with MIDOC trawl net	Volume Filtered = (1) Distance towed (m) * (2) the net’s filtering area (m^2^)	Ship’s GPS position at the start and end of trawl	headline height × wingspread
ANT21_4_PS65	RMT1 + 8	Mechanical Flow Meter - Hydrobios		
Lakris Summer: ANT23_2_PS69_4	RMT8	Mechanical Flow Meter - Hydrobios		
Lakris Winter: ANT23_6_PS69_6	RMT1 + 8	Mechanical Flow Meter - Hydrobios		
KARE 22	MOHT	Volume Filtered = (1) Distance towed (m) * (2) the net’s filtering area (m^2^)	Mechanical flow meter on MOHT frame	0.7355 × 5 (m^2^) (0.7355 is the coefficient from calibration tows in calm seas and 5 (m^2^) is the net mouth opening)
Ichtyoker	IYGPT	Volume Filtered = (1) Distance towed (m) * (2) the net’s filtering area (m^2^)	Tow duration (min) * Speed (m/min)	headline height × wingspread
US AMLR Program	IKMT	General Oceanics flow meter (model 2030 R) mounted on the depressor frame in front of the net		

*Calculated retrospectively as outlined in Data Collection and Processing.
